# A Plea for Sanatoria for the Poorer Consumptives

**Published:** 1900-12

**Authors:** Lionel A. Weatherly


### A PLEA FOR SANATORIA FOR THE POORER CONSUMPTIVES.

BY

LIONEL A. WEATHERLY, M.D.

---

"Is there anything whereof it may be said, See this is new?"

IN 1882, Koch announced to the world his discovery of the bacillus tuberculosis, and proved that consumption was a communicable disease.

Four hundred years before Christ, Isocrates preached the doctrine of the contagiousness of phthisis; but few listened to him.

Half a century A.D., Pliny the Elder prescribed exposure to the sun and residence in the neighbourhood of pine forests for those afflicted with consumption.

In 1840, an English country practitioner, Dr. Bodington, of Sutton Coldfield, advocated and carried out the very treatment of consumption which to-day is looked upon as the best, the most common-sense, and most hopeful mode of helping forward recoveries in the early stages of this disease. Dr. Bodington's ideas met with the bitterest and most violent opposition, and dissent was so strongly expressed by the general body of the medical profession that he was neither able to pursue his investigations, nor carry out the practice of his treatment to any great extent.

In 1856, Dr. Herman Brehmer wrote his celebrated thesis on *Consumption and its Curability*, and three years later obtained, after considerable difficulty, authority to build a sanatorium for his good work at Goerbersdorf.

In 1874, Dr. Dettweiler, his pupil, with the help of a few of Frankfort's wealthy citizens, opened the celebrated institution for the treatment of consumption for the wealthier classes, at Falkenstein, and from the profits thereby gained there is to-day at Ruppertshain, close by, one of the best sanatoria for the poor in Germany. It may be safely affirmed, that to the labours of Brehmer and Dettweiler, to the success of their untiring energies and individual supervision, we owe to-day the universal adoption of the most successful mode of treatment of a disease, the very name of which had always been looked upon as a death sentence to so many of our brightest, our cleverest, our most loveable.

Gradually, but slowly, the doctrines of this common-sense and rational treatment have been practised all over the civilised world, and in many countries have been acted upon with considerable success. In England, until recently, an unaccountable apathy has pervaded the minds of the people ; and from being once in the forefront of charitable institutions for her consumptives, she has been gradually drifting far behind. The National Association for the Prevention of Consumption has done a great deal to stir up interest, if not enthusiasm, in our land, and we are hoping for still more help from the International Congress on Tuberculosis, which is to take place next year in London, under the presidency of H.R.H. the Prince of Wales.

Though lay writers, in a lay press, have also done much to bring the new method of treatment before the English public, I fear, from too great enthusiasm, they have been led to be far too superlative in the recount of their "experiences," and thus have done a certain amount of real harm. To build up extravagant hopes of wholesale cures in a short time, can only handicap most seriously the adoption of the most successful treatment of any dangerous and deadly disease. The only true and right way to allow a new idea of treatment to be universally applied, and to assure a general active interest and co-operation in the modes of carrying it out, more especially among the poorer classes, is to make the public definitely understand the *rationale* of its application, and the large amount of good it must do, not only by curing certain cases, but, what is still of equal importance, by preventing the spread of a disease which science now tells us, with no uncertain voice, can in time be rooted out.

In what then does this new treatment consist? What may we reasonably hope from it? How can we best enable our poorer classes to obtain the benefits to be derived from its application?

Luckily, there is nothing controversial about it. It does not emanate from the brains of a few enthusiastic faddists; neither is it the idea of money-making quacks. It is supported by the most eminent and most scientific medical men in the civilised world. It has already proved beyond contention its great value. It is doubtful whether the name "open-air treatment" is comprehensive enough, though it may be difficult to find a more suitable one. Some have suggested "hygienic treatment," but this does not, I think, sufficiently convey to the lay mind the salient features of its methods.

Generally speaking, we may say that the rules by which we hope to successfully combat many cases of consumption are the following:—

Fresh pure air, and an abundance of it, not only by day, but also by night; a generous dietary, both in quality and quantity; properly regulated exercise in the open air, and properly regulated rest; a gradual training whereby the patient is able to live out of doors in almost all weathers, without fear of catching cold; freedom as far as possible from mental worry; and, lastly, *unremitting medical supervision.*

The individual medical supervision is indeed of the utmost importance. Plenty of fresh air, plenty of good food, will avail but little if the daily life of the patient is to be left to his own willing, and he is allowed to "do as he pleases." In an article on this subject in the *Year-Book of Treatment* for 1899, Dr. Burton Fanning emphasises this point: "Hitherto we have failed in our management of phthisical cases through lack of insistance and perseverance. In our heart of hearts we have been inclined to regard the disease as an intractable one, and we have therefore had insufficient confidence in our treatment of it. The patient has not been convinced that particular rules are worth following to the letter, and that the difference between recovery and permanent ill-health may depend on whether he devotes himself absolutely to a certain mode of life or follows it in a haphazard, intermittent fashion. Recognising the difficulty of enforcing a *régime* which is antagonistic both to the patient's prejudices and former habits, without removing him from home, so far, this treatment has been mostly carried out in large sanatoria."

The open air purifies the blood, promotes appetite, and aids assimilation; the general treatment and the rules and regulations laid down by the thoughtful medical man, who has studied the individualities of his patient, fortifies the system to resist the disease.

In carrying out the treatment to the fullest extent, and in order to give the patient the greatest chance of recovery, situation and surroundings are important factors. We need not insist upon any definite climate, for it has now been pretty well proved that climate exerts only a secondary influence on results. But it is essential that the air should be pure, and free from dust; that the soil should be dry; and that the situation should be of such a character that the patient may be able to sit out in the open air, and take regulated exercise. It is therefore self-evident that numberless consumptive patients cannot be properly treated in their own homes, and more especially is this the case among our poorer classes; and the stern necessity for some suitable place to which our poorer consumptives can be sent for treatment becomes at once clear. Sanatoria for the richer classes are springing up in all directions, but so far little has yet been systematically done in England towards the erection and maintenance of these institutions for the poor; though we all ought to recognise, as a writer in the *British Medical Journal* pointed out, with regard to consumption among the poor, that "their poverty prevented their isolation, their ignorance caused neglect of precautionary measures, and their surroundings increased so greatly the susceptibility to infection."

Take the large city of Bristol. Realise for a moment the number of consumptives belonging to the poorer portion of the community, and ask yourselves, How can they at the present time be properly treated? Will your hospitals admit them? And if they do, can they be isolated, can they be treated in the manner I have already described, and are the situation and surroundings at all suitable? Can they possibly hope to obtain any real benefit from attending the out-patient department? Can they be treated with any reasonable hope of success in their own homes? Where are they then to obtain treatment, where can they hope for some chance of escaping from their otherwise inevitable doom, and how can they be prevented from disseminating the seeds of their frightful disease, if we do not provide an institution specially adapted by its site, its surroundings, and its management, not only for their care, but also for their cure?

I do not intend to burden this short article with statistics, nor do I desire it to be thought that I believe that the open-air treatment is a sure and certain remedy for this terrible disease; but I do most emphatically say, that in the history of medicine no other form of treatment of consumption has ever been in any degree so successful, and that, provided the patient be placed under its influence in the very early stage of the disease, the percentage of "arrest" or "recovery" is very high.

When we realise that in England and Wales alone some 60,000 die each year of this complaint; when we hear, that in one large city of England last year, every time the clock registered its four hours a death from consumption was taking place; and when we are told by science and practical experience that many of those lives might have been spared had they been treated by these new methods, ought we to hesitate a moment to do all in our power to give everyone under the direful influence of this insidious and dreaded disease the only means whereby they may be enabled to resist its ravages?

In Germany, where the open-air sanatoria for the poorer classes have lately been so largely established, it is roughly estimated that about 30 per cent. can be reported as "cured," and that another 40 per cent. can be marked down as "greatly improved." These results are not in any way due to the influence of climate, but simply to the treatment already described. Knowing all this, is it not our bounden duty to use all the appliances science has given us to bring about what some will call an "arrest," others will designate by the more cheering name of "recovery," but what, call it as you may, is in numberless cases a priceless boon?

How then can we hope to start such institutions, how can we expect to maintain them? I will at once state my very firm opinion that the Boards of Guardians all over our country, either separately or in combination, ought to provide suitable places in which the rate-supported consumptive could be properly treated, *and that this ought not to be left to private charity.* For the classes above these, we cannot expect, I fear, any help from Local Government, and we must look to the good and kind feelings of those who have been blessed with the wherewithal to give.

To build and equip a sanatorium for sixty patients, not counting the land, ought not to cost more than £200 a bed. The simpler the building, the better, and decorations should be conspicuous by their absence. It is clear we shall have to look to private philanthropy to enable us to build such institutions in certain districts of our land. Already some have been started, and generous donors of large sums have come forward to materially lessen the difficulty of raising sufficient funds, and I do not for one moment believe that our West of England will allow itself to be left behind in this good work. To maintain such an institution would, I think, cost between £60 or £70 per bed per annum. I know some of the recently started sanatoria for the poor have reckoned on a lesser sum than this; but I fear they will find that the expense of the liberal dietary, so essentially necessary, will prove their figures to be under the mark.

How then can such an annual sum be raised? I would first of all suggest that clubs and friendly societies should amalgamate in their respective districts, and should subscribe a certain sum yearly, having in return a claim of beds according to the amount of their subscriptions. Factories and large works might do the same, and I believe Town Councils and County Councils have it in their power, or could obtain power, to subscribe to such a good cause. Then those patients seeking admission, who could not procure a ticket from any of these bodies, should either pay themselves a small sum per week, or get some of their friends to help them, as is the case at present in most of our convalescent homes. The balance required to meet the expenses of maintenance should be readily collected from the district to which the sanatorium belongs.

It must be generally understood that these institutions are only for recoverable cases, and it should be strongly urged upon all medical men to do their utmost to send the cases at the earliest possible moment after diagnosis of the disease. The average length of time in the sanatorium to be allowed each case should, I think, be left in the hands of the committee managing the institution, who would of course be guided by their medical superintendent; but we may reasonably hope that a sanatorium for sixty beds would be able to treat at least 120 to 150 cases per year, and more even than that, if only cases in the very early stage were sent there, and if some after-care association could be started in conjunction with the sanatorium. This is rather beside my subject, but its importance must strike everyone who has taken any interest in this treatment, and might form the basis for another paper.

To take a case of early phthisis from the close atmosphere of a factory or an office, and from the unhealthy dwelling-house in a crowded thoroughfare, to cure or arrest the disease, and then to send the case back again to the same atmosphere, must inevitably mean relapse. We hear a great deal about the moving of the rural population into towns, about the difficulty the farmers of our country have to get helpers in their work. May we not here find a partial solution of one of the greatest difficulties in the after-care of our so-called "cured consumptive"?

In Germany, all persons directly they enter on a career as labourers or servants have to insure against sickness, accident, and old age. These State Insurance Companies have very materially helped forward the building and the maintaining of the sanatoria for the poor, and directly consumption develops in any of the members of these companies, the case is at once sent to one of the sanatoria. Dr. Weicker, of Goerbersdorf, to whose institution a large number of such patients are sent, informed Dr. Knopf, whose work on *Pulmonary Tuberculosis* is of the deepest interest, that the percentage of cures among these is higher than among the private patients. His latest statistics give a percentage of 80 established cases with only an average of 76½ days' sojourn in the sanatorium. This marvellous result proves how vitally important it is to send the consumptive for definite treatment at the earliest possible period of the disease.

Sir James Crichton Browne, whose friendship I value and whose ability I admire, and whose interest in the establishment of sanatoria for the poor is so great, recently declared that he believed if the public realised what could be done by united effort to stamp out the disease, England might be freed from its grip in, comparatively speaking, some seventy-five years. A very utopian idea, some of you may say, and yet there may be more truth in it than at first appears.

Pasteur has told us that "it is in the power of man to cause all parasitic diseases to disappear from the world." The worst of all parasites is that discovered by Koch, the bacillus tuberculosis. To exterminate this enemy from our midst, we must first cure our curable consumptives, while at the same time we are teaching the public the means whereby they may prevent any further spread of the disease. To do this, nothing is more essential than the introduction of properly situated, properly equipped, and properly managed sanatoria for our rich and our poor. The rich have the remedy in their own hands, and already have large numbers of such places both in this and other countries to choose from; but for those who cannot help themselves, it becomes the duty of all to do their very utmost to help forward a movement, by which each district in our land shall have such an institution for the treatment of its poorer consumptives.

To carry this good work to a successful issue, united effort is needed; enthusiasm, like the disease we wish to banish, must be made contagious; the goodwill of an intelligent people must be awakened; and, finally, we must be able of each one of the wealthy in our midst, of a truth to say—

"He hath a tear for pity, and a hand
Open as day for melting charity."

